# 751 Burn Therapy Certified Therapists Representation within Burn Society Leadership

**DOI:** 10.1093/jbcr/irad045.226

**Published:** 2023-05-15

**Authors:** Audrey O'Neil, Derek Murray, Renee Warthman

**Affiliations:** Richard M. Fairbanks Burn Center, Indianapolis, Indiana; Arizona Burn Center Valleywise Health, Phoenix, Arizona; Arizona Burn Center at Valleywise Health, Phoenix, Arizona; Richard M. Fairbanks Burn Center, Indianapolis, Indiana; Arizona Burn Center Valleywise Health, Phoenix, Arizona; Arizona Burn Center at Valleywise Health, Phoenix, Arizona; Richard M. Fairbanks Burn Center, Indianapolis, Indiana; Arizona Burn Center Valleywise Health, Phoenix, Arizona; Arizona Burn Center at Valleywise Health, Phoenix, Arizona

## Abstract

**Introduction:**

The American Burn Association (ABA) Burn Therapist Certification (BT-C) program, developed in 2018, derived from an organizational desire to promote vetted quality burn care across the interprofessional team. Leadership positions within organizations, are responsible for formulating policy, developing education, and providing oversite to ensure the success of the association. A previous study examining interdisciplinary representation within burn society leadership for the ABA and International Society of Burn Injuries, found that rehabilitation representation within governance positions was 7% and 9% within committee memberships. In a 2022 Letter to the Editor, therapists described limited center support as a barrier for obtaining BT-C. This study aims to examine BT-C member involvement within ABA society leadership.

**Methods:**

Official ABA society and membership websites were accessed to identify BT-C recipients who currently hold positions within society governance and committee memberships. ABA Special Interest Group (SIG) membership was also examined. The Journal of Burn Care and Research (JBCR) website was accessed to identify BT-C recipients on the editorial board. BT-C members were compared to other therapist involvement.

**Results:**

Of 32 qualified BT-C therapists, 26 continue their ABA memberships. BT-C therapists hold 3 Governance positions (Board membership and current ABA President) and maintain 36 of the available 77 committee positions (47%). Excluding the BT-C Committee from data collection, BT-C therapists make up 44% (n=23) of therapist committee positions within the ABA. BT-C therapists hold a combined 46 SIG memberships. The JBCR currently lists 2 therapists on the editorial board, both of whom are BT-C.

**Conclusions:**

Despite representing a fraction of the burn therapy community, BT-C therapists continue to be leaders within the burn field by participation in ABA membership, governance, and committee memberships. Current BT-C therapists serve to establish standards in research published within the JBCR. Professional and academic societies depend on qualified leaders to set standards of burn care. Promoting BT-C within individual burn centers may stimulate leadership within the center and burn professional organizations.

**Applicability of Research to Practice:**

Definitive evidence supporting the benefits of BT-C to individuals and organizations is needed to overcome current barriers to certification.

